# FSH/LH co-stimulation in Advanced Maternal Age (AMA) and hypo-responder patients – Arabian gulf delphi consensus group

**DOI:** 10.3389/fendo.2024.1506332

**Published:** 2024-12-12

**Authors:** Johnny Awwad, Braulio Peramo, Bohaira Elgeyoushi, Laura Melado, Anastasia Salame, Monika Chawla, Salam Jibrel, Sajida Detho, Hazem Al Rumaih, Mustapha Tomsu, Khaled Fahim, Mohamed Abd-ElGawad, Alaa Fouad, Peter Humaidan

**Affiliations:** ^1^ Women’s Services and Reproductive Medicine and IVF Center, Sidra Medicine, Doha, Qatar; ^2^ Obstetrics, Gynecology, and Reproductive Medicine, Al Ain Fertility Center, Al Ain, United Arab Emirates; ^3^ Obstetrics and Gynecology, Dr Sulaiman Al Habib Fertility Centre, Dubai, United Arab Emirates; ^4^ ART Fertility Clinics, Abu Dhabi, United Arab Emirates; ^5^ ART Fertility Clinic, Al Ain, United Arab Emirates; ^6^ Reproductive Medicine, Health Plus Fertility Center, Abu Dhabi, United Arab Emirates; ^7^ Salam IVF Center, Arabian Gulf University, Manama, Bahrain; ^8^ Bournhall IVF Centre, Al Ain, United Arab Emirates; ^9^ Obstetrics and Gynecology, New Jahra Hospital, Ministry of Health, Kuwait City, Kuwait; ^10^ Reproductive Medicine, Tomsu Fertility Clinic, Salmiya, Kuwait; ^11^ Medical department, Merck Serono Middle East FZ-LTD, Dubai, United Arab Emirates; ^12^ Preventive Medicine, Ministry of Health & Population, Fayoum, Egypt; ^13^ The Fertility Clinic, Skive Regional Hospital, Skive, Department of Clinical Medicine, Aarhus University, Skive, Denmark

**Keywords:** LH, FSH, gonadotrophins, poor responders, poor ovarian reserve, advanced

## Abstract

**Background:**

In a global effort to assess expert perspectives on the use of recombinant gonadotropins, recombinant human luteinizing hormone (r-hLH) and recombinant human follicle-stimulating hormone (r-hFSH), a consensus meeting was held in Dubai. The key aim was to address three critical questions: What are the factors that influence follicle response to gonadotropins? Which categories of patients are most likely to benefit from LH supplementation? And what are the optimal management strategies for these patients?

**Methods:**

A panel of thirty-six experts reviewed and refined the initial statements and references proposed by the Scientific Coordinator. Consensus was defined as agreement or disagreement by more than two-thirds (66%) of the panel members for each statement.

**Results:**

Thirty-five statements were formulated, of which thirty-one reached consensus. For patients with Hypo-Response to Gonadotropin Stimulation (20 statements), all identified risk factors, including advanced age, high BMI, and chronic conditions, achieved unanimous agreement. Diagnostic approaches, such as the inclusion of POSEIDON criteria and hormone level monitoring, were endorsed by the majority, with over 90% agreement. Management strategies, particularly individualized stimulation protocols and optimized scheduling, garnered broad consensus, with only one statement falling short of the threshold. Additionally, in cases of severe FSH and LH deficiency, combining r-hFSH with r-hLH was found to improve pregnancy rates and cost efficiency compared to human menopausal gonadotropin (hMG). For patients with Advanced Maternal Age (AMA) (15 statements), there was strong agreement on the use of oral contraceptive pills and estrogen priming. Recommendations concerning antagonist protocols and dosing of r-hLH and r-hFSH also achieved high levels of consensus. Significant agreement supported r-hLH supplementation and a tailored approach to luteal phase support. However, there were mixed opinions on the route of progesterone administration, with some experts expressing neutral or disagreeing views. Despite these differences, unanimous consensus was reached on markers of treatment success, particularly live birth rates, pregnancy rates, and embryo development, underscoring the importance of these outcomes in evaluating treatment efficacy.

**Conclusion:**

This consensus provides a practical clinical perspective to a wide range of global professionals on the strategies employed during key phases of Assisted Reproductive Technology (ART) treatment. To further improve outcomes, incorporating additional clinical insights on ART approaches, alongside existing guidelines and policies, may offer valuable guidance for optimizing patient care.

## 1 Introduction

Infertility stands as a substantial healthcare challenge globally (1, 2), with one in six couples encountering at least one reproductive issue during their fertile years (3). The sustained evolution of assisted reproductive technology (ART) positions it as a powerful solution for addressing infertility (4). Ongoing progress in this area has led to the development of more advanced diagnostic tools and various treatment options, making assisted reproductive procedures more effective (5, 6).

A successful response to ovarian stimulation (OS) is vital for the effectiveness of ART (7). The quantity of retrieved oocytes is commonly used to evaluate how well the ovaries respond to external gonadotropins, and it closely correlates with the live birth rate (8). Women are typically categorized as poor, normal, or hyper-responders based on ovarian biomarkers and the number of oocytes (7, 9). However, there exists a specific group known as “hypo-responders,” characterized by an unexpectedly inadequate response to gonadotropin therapy despite having suitable pre-stimulation ovarian parameters (10, 11).

The factors contributing to hypo-responsiveness to gonadotropin stimulation are not entirely understood, but they may be linked to genetic mutations of gonadotropin receptors (12, 13). Patients with this condition exhibit an unforeseen resistance of the ovaries to OS despite receiving standard doses of exogenous gonadotropins tailored to their age and BMI (10, 13). Ovarian resistance can be clinically indicated by either an “initial slow response” to FSH stimulation observed through a gradual increase in estradiol levels and follicle growth (14, 15), or it can be identified retrospectively in women needing doses of gonadotropins higher than anticipated, accounting for their age, BMI, and ovarian reserve (16).

Based on the above-mentioned data and for more clarification, hypo-responsiveness to gonadotropins can be classified into two main categories: advanced maternal age and hypo-responders. Advanced maternal age typically includes women aged 35 or older, who commonly experience a decline in ovarian function, leading to a reduced response to gonadotropin stimulation due to factors such as diminished ovarian reserve and oocyte quality (17). Additionally, elevated BMI, particularly in the overweight or obese range, is associated with hormonal imbalances and altered ovarian function, resulting in a diminished response to gonadotropin therapy. Furthermore, women with markers indicating diminished ovarian reserve, such as low antral follicle count (AFC), high basal FSH levels, or low anti-Müllerian hormone (AMH) levels, are also expected to have a poor response to gonadotropin stimulation due to a reduced pool of recruitable follicles (16).

On the other hand, hypo-responders comprise women who, despite having characteristics typically associated with a robust ovarian response, such as younger age, lower BMI, and normal ovarian reserve markers, unexpectedly exhibit a poor response to gonadotropin stimulation (11, 13). This subgroup may include individuals with underlying genetic factors affecting ovarian function, undiagnosed medical conditions such as polycystic ovary syndrome (PCOS), endometriosis, or thyroid disorders, or lifestyle factors such as smoking or environmental exposures (13, 18). Additionally, some women may have unexplained factors contributing to hypo-responsiveness, necessitating further investigation to determine the underlying cause. This may include assessing for factors such as uterine abnormalities, immune factors, or psychological stressors impacting ovarian function (19, 20).

The primary challenge in OS hyper-responsiveness cases is the inconsistency between the number of retrieved oocytes and the ovarian reserve. A more “potent” gonadotropin formulation is recommended to enhance the initial oocyte retrieval during stimulation. Numerous trials and pooled analyses have indicated that recombinant preparations result in more retrieved oocytes than urinary preparations (21–23). These observations appear to be associated with the higher bioactivity of recombinant preparations (24).

Urinary-derived human gonadotropins, the exclusive commercially available gonadotropins for more than three decades (19), faced challenges such as low purity, impurities, and fluctuating levels of FSH and LH (25, 26). In contrast, recombinant FSH (r-hFSH) offers a highly pure (>99%) and potent alternative with minimal contamination. Studies suggest that the purest r-hFSH may be preferred for hypo-responsiveness to gonadotropin. Recombinant technology ensures high purity, low immunogenicity, and independence from urine collection, positioning it as an advanced and reliable option (27).

A new approach for OS has been suggested for women with a low prognosis, aiming to enhance the number of retrieved oocytes and the availability of blastocysts for biopsy in a single ovarian cycle (28). In this strategy, patients are co-treated with a maximum dosage of r-hFSH at 300 IU/day, along with r-hLH at 150 IU/day for both follicular and luteal phase stimulation (29).

In clinical settings, the insufficient response to gonadotropin stimulation, termed hypo-responsiveness, is often overlooked. Clinicians typically do not assess whether the number of oocytes retrieved during ovarian stimulation aligns with the patient’s potential, considering the initial AFC results. Limited research has been dedicated to interventions for women experiencing ovarian resistance (hypo-responsiveness) during ovarian stimulation, and until recently, there were no available practical guidelines. To bridge this gap, a consensus meeting in Dubai was convened to gather and assess global expert opinions on the use of r-hFSH and r-hLH during critical stages of ART treatment. The objective was to build on the existing evidence from the European Society of Human Reproduction and Embryology (ESHRE) guidelines by incorporating expert opinions, offering additional clinical insights to refine treatment strategies and enhance patient outcomes.

## 2 Materials and methods

### 2.1 Sponsorship

The Gulf consensus was facilitated by a healthcare consulting and training company Mind Leap Educational Services LLC. Merck Serono Middle East FZ-LTD initiated and funded the consensus concept. While the sponsor played a role in the early stages, defining the overarching topic, they did not contribute to formulating statements or partake in subsequent meetings or discussions throughout the development of the Gulf consensus. Consequently, the statements were independently crafted without the influence of an industry sponsor. Merck’s involvement was limited to manuscript development, specifically in the Introduction and Results sections. However, they had no authority to modify the consensus statements.

### 2.2 Participants

The Gulf consensus began with a Scientific Board staffed by ten leading experts in the field.

### 2.3 Methodology

**Figure 1:**
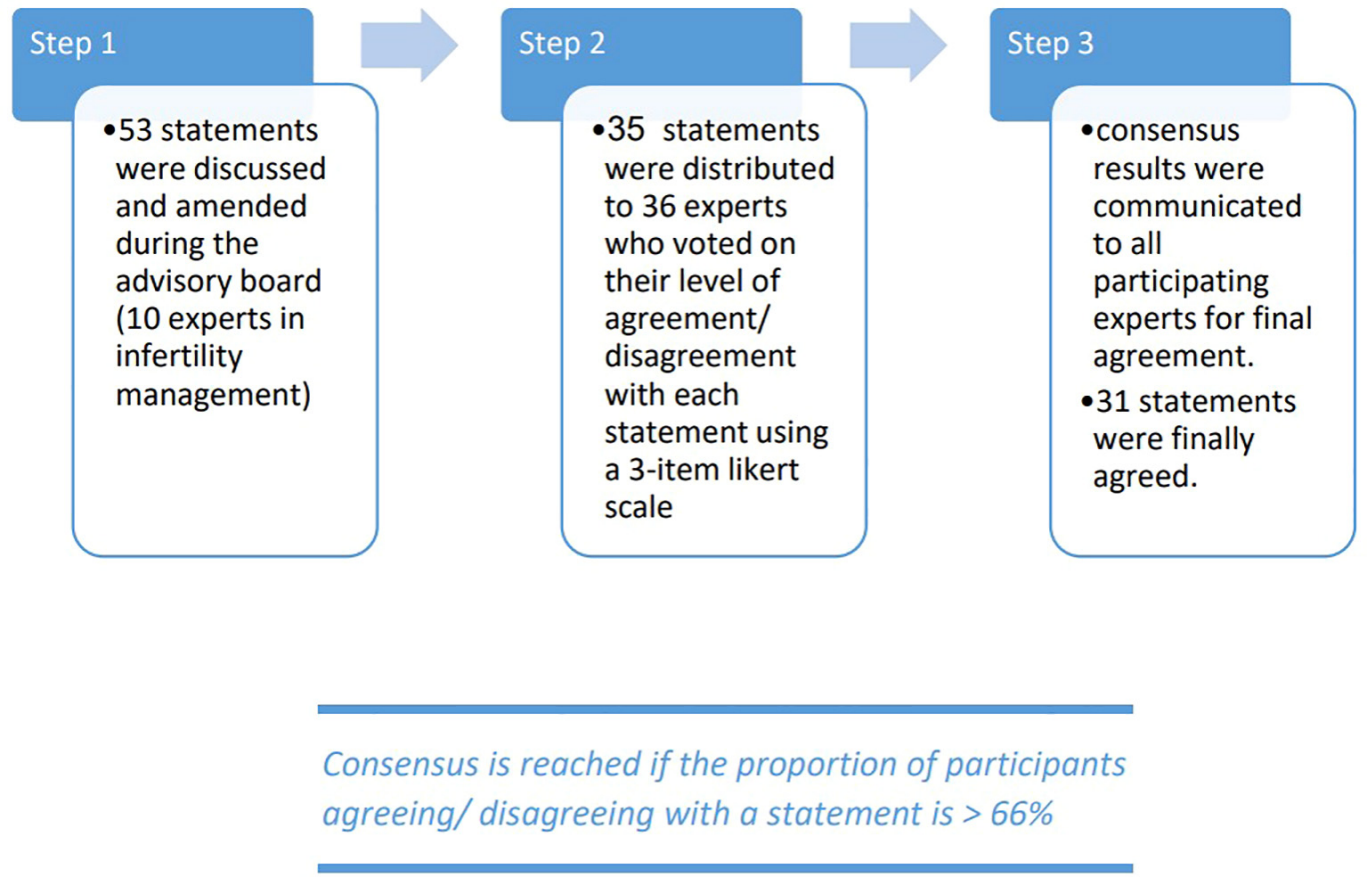
Three stages of the gulf consensus.

The Gulf consensus process was conducted in three stages (**Figure 1**). Initially, the Scientific Coordinator developed 53 statements with supporting references based on a comprehensive review of the latest scientific literature. Step 1 involved the Scientific Board, who reviewed these statements in two web conferences, where they had the authority to revise, add, or remove statements and references. Through consensus among the Scientific Coordinator and the Board, a final set of 35 statements and associated references was established for the next stage. Step 2 aimed to gather consensus from the Extended Panel on the statements refined in Step 1. An online survey was distributed to the Extended Panel (comprising 26 external experts) and the 10 core experts, asking each participant to anonymously rate their agreement using a three-point Likert scale. A statement was considered to have reached consensus if more than 66% of participants either agreed or disagreed with it. Step 3 involved the collection and analysis of the survey results, with 31 statements ultimately achieving the 66% agreement threshold.

## 3 Results and discussion

**Figure 2:**
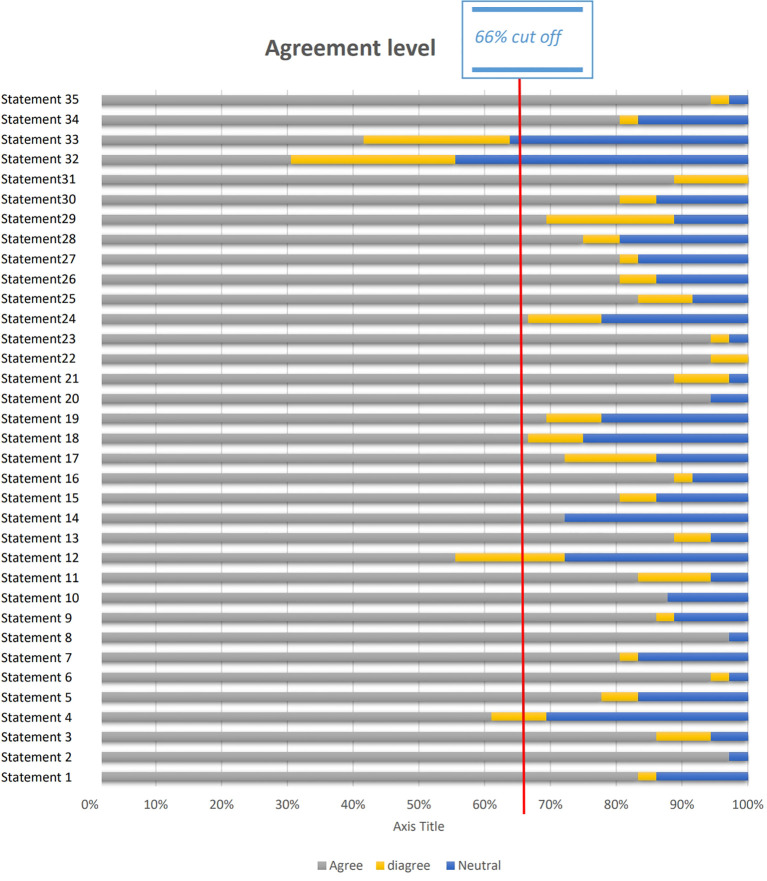
Outcomes of the consensus.

The results are divided into two main categories (1): Patients with Hypo-Response to Gonadotropin Stimulation (20 statements): These included statements on patient profile at presentation (2 statements), diagnostic tools (3 statements), management techniques (14 statements), and markers of success (1 statement) (2); Patients with Advanced Maternal Age (AMA) (15 statements): These statements addressed patient profile at presentation (2 statements), diagnostic tools (3 statements), management techniques (9 statements), and markers of success (1 statement). In total, 35 statements were presented. Of these, 31 achieved consensus (≥66% agreement), indicating strong alignment on critical factors for managing hypo-response to gonadotropins and AMA in fertility treatments (**Figure 2**).

### 3.1 Patients with hypo-response to gonadotrophins stimulation

#### 3.1.1 Profile at presentation

##### 3.1.1.1 Statement 1

Consensus identified several risk factors that contribute to hypo-response to FSH, including advanced age (>35), high BMI (>30), consanguinity, variations in ethnicity, smoking, environmental pollution, chronic conditions (such as hypothyroidism or uncontrolled diabetes mellitus), and long-term medications—particularly those that may enhance hepatic metabolism or affect the central nervous system (CNS). This statement achieved an agreement rate of 83.33%.

##### 3.1.1.2 Discussion

The panel discussed the interaction between aging and various clinical and therapeutic factors, emphasizing that these elements can influence gonadotropin action and the body’s response to exogenous gonadotropins, thereby affecting the effectiveness of FSH (30). As women age, their ovarian reserve declines, resulting in diminished responsiveness to FSH stimulation, a phenomenon particularly significant for those over 35 years old. This age-related decline in ovarian function has been thoroughly documented in numerous studies, reinforcing the consensus that age is a critical determinant of fertility outcomes (30–32).

It was agreed that a high BMI, particularly when exceeding 30, is linked to altered hormonal profiles, including decreased sensitivity to FSH. Adipose tissue produces hormones that can disrupt the hypothalamic-pituitary-gonadal (HPO) axis (33). A prior study examining the relationship between elevated BMI and ovarian response to FSH stimulation indicated that managing obese non-PCOS patients with hypogonadotropic response presents greater challenges (34).

It was recognized that consanguineous marriages (between close relatives) may elevate the risk of genetic mutations that impact gonadotropin receptors or signaling pathways. Although specific studies examining the relationship between consanguinity and FSH response are limited, it is acknowledged that genetic factors influence gonadotropin action (35). Additionally, genetic and epigenetic differences among ethnic groups can affect the expression and function of gonadotropin receptors. While the ethnic variations in gonadotropin action have not been extensively researched, they are noted in the existing literature (36).

Smoking negatively impacts ovarian function, potentially resulting in decreased responsiveness to FSH. Nicotine and other components of tobacco can also disrupt hormonal balance (37). Although the relationship between smoking and hypo-response is not directly established, its detrimental effects on fertility are well-documented across various studies (38–40). Similarly, exposure to environmental toxins, including endocrine-disrupting chemicals, has been linked to impaired fertility and altered hormonal regulation, which may contribute to reduced responsiveness to FSH (41).

The panel also recognized that ovarian function is closely tied to overall health, with chronic diseases having a significant impact on ovarian responsiveness, potentially resulting in hypo-response. Hypothyroidism, marked by inadequate thyroid hormone production, can disrupt the hypothalamic-pituitary-ovarian (HPO) axis, leading to irregular menstrual cycles and ovulatory dysfunction. Thyroid hormones are essential for ovarian folliculogenesis and steroidogenesis, and any alterations in thyroid function can adversely affect ovarian responsiveness to hormonal stimulation (42, 43). Additionally, uncontrolled diabetes mellitus, particularly type 2 diabetes, is linked to insulin resistance and hyperglycemia, both of which can negatively impact ovarian function. Hyperglycemia-induced oxidative stress and inflammation may damage ovarian tissues and disrupt follicular development, ultimately leading to diminished responsiveness to FSH stimulation (44).

The experts acknowledged that certain long-term medications, such as antiepileptics and antidepressants, may affect gonadotropin receptors or hormone production. These medications can disrupt hormonal balance and metabolic regulation, subsequently impacting ovarian reserve and fertility potential (45, 46). Although specific studies focusing on hypo-response are limited, it is essential to understand the effects of these medications in the context of assisted reproduction.

##### 3.1.1.3 Statement 2

The panel reached a consensus that ovarian function can vary in response to hypo-response, which may be influenced by receptor polymorphisms and genetic variations, as well as chronic conditions such as hypothyroidism and uncontrolled diabetes mellitus. This statement garnered an agreement rate of 97.22%.

##### 3.1.1.4 Discussion

Greb et al. highlight the ongoing gap in research regarding the therapeutic applications of pharmacogenomics in Assisted Reproductive Technology (ART) (47). However, emerging data suggest that ovarian response to exogenous gonadotropins may be influenced by specific genetic traits related to gonadotropins and their receptors. For instance, increased FSH consumption during controlled ovarian stimulation (COS) has been associated with the FSH receptor (FSHR) single nucleotide polymorphism (SNP) rs6166, indicating a potential effect on ovarian responsiveness (48). Additionally, this SNP has been correlated with elevated basal FSH levels, which may signify compromised responses to both exogenous and endogenous gonadotropins (49–51). Furthermore, a poor ovarian response has been linked to another FSHR polymorphism, rs1394205, as reported by Achrekar et al. (52).

Similarly, individuals with a SNP in the gene encoding the LH beta subunit have shown suboptimal responses to *in vitro* fertilization (IVF). Previous studies have emphasized the influence of LH receptor SNPs, specifically LHCGR rs2293275 and LHCGR rs12470652, on controlled ovarian stimulation (COS) and Assisted Reproductive Technology (ART) (53, 54). These findings suggest that a “hypo-response” to gonadotropin therapy may be linked to specific genotype characteristics. Furthermore, they underscore the importance of genetic biomarkers, particularly those associated with FSH and its receptor, in predicting and optimizing ovarian response to gonadotropin stimulation in ART contexts (49).

Ovarian function is intricately connected to overall health, with chronic illnesses significantly impacting it. Thyroid dysfunction, the most common endocrine disorder among women of reproductive age, serves as a notable example. Both overt and subclinical hypothyroidism are associated with female infertility, leading to issues such as anovulation and irregular menstrual cycles (55, 56). In light of these findings, the administration of thyroxine supplements has become a standard practice for women attempting to conceive when their thyroid-stimulating hormone (TSH) levels are below 2.5 μIU/mL (57). However, research on this TSH cut-off has yielded inconsistent results (58–60). Reh et al. reported that IVF patients with TSH cut-offs of 2.5 μIU/mL and 4.5 μIU/mL exhibited similar rates of pregnancy and delivery among euthyroid individuals (61). In contrast, Murto et al. identified that a TSH level of less than 2.5 μIU/mL and an AMH level of 10 pmol/L or higher were significant predictors of live births in women with unexplained infertility (62). While most patients typically maintain TSH values below 2.5 μIU/mL around the time of ovulation, TSH levels can increase by 50–80% from the beginning of the cycle until ovulation induction with hCG during controlled ovarian stimulation (COS). Notably, individuals with an initial TSH level exceeding 3.0 μIU/mL at the start of the cycle face a significant risk of developing severe clinical hypothyroidism (63, 64).

Various unknown factors influence the impact of impaired thyroid function on female fertility. It has been proposed that thyroid dysfunction may hinder follicular growth and maturation, as evidenced by documented associations with irregular menstruation and anovulation in hypothyroidism (65). Based on this premise, AMH concentrations are expected to be affected by TSH levels, irrespective of a woman’s age or thyroid autoimmunity. A recent study by Busnelli et al. demonstrated that TSH levels increase during ovarian stimulation, and women undergoing fertility treatments with TSH levels greater than 2.5 μIU/mL tend to exhibit lower AMH concentrations (66).

The expert panel agreed that uncontrolled diabetes mellitus can also negatively impact ovarian function. Research by Codner et al. indicates that approximately 40% of women with type 1 diabetes (T1DM) experience irregular menstruation, hyperandrogenism, or infertility. In women with T1DM, these symptoms may manifest early in their reproductive lives due to the complete absence of insulin. Insulin has been shown to enhance menstrual cycle regularity and protect against infertility (67). However, in individuals with T1DM, insulin resistance can lead to additional complications, including hyperandrogenism and polycystic ovary syndrome (PCOS). Although these conditions are commonly observed in premenopausal women with T1DM, it is crucial to note that there is a lack of available data on this topic, and the underlying pathophysiology of this association remains poorly understood (68). In contrast, type 2 diabetes mellitus (T2DM) is characterized by insulin resistance (IR) and elevated insulin levels (hyperinsulinemia). This IR has been linked to an increased risk of menstrual irregularities, infertility, and the development of polycystic ovary syndrome (PCOS) in women with T2DM. The disruption of normal ovarian function in T2DM occurs because IR and hyperinsulinemia promote the excessive production of insulin-like growth factor 1 (IGF-1) and the overexpression of hybrid insulin/IGF-1 receptors. This stimulation affects ovarian granulosa cells, leading to abnormal follicle development and the enlargement of ovaries containing multiple small follicles. The overactivity of IGF receptors contributes to elevated ovarian androgen production, as insulin continues to influence ovarian function, resulting in clinical hyperandrogenism in women with T2DM. Additionally, high insulin levels mimic gonadotropins’ action on theca cells in the ovaries, inhibiting the recruitment of a dominant follicle and causing menstrual disturbances and anovulation (69).

#### 3.1.2 Diagnostic tools

##### 3.1.2.1 Statement 3

Patients with a history of a low follicle-to-oocyte index (FOI) of less than 50% in previous cycles, despite having normal AMH levels, should be managed by incorporating r-hLH from the onset of treatment or using it as a rescue option if identified later in the cycle. A significant majority, 86.11%, agreed with this approach.

##### 3.1.2.2 Discussion

Patients with a history of low follicle-to-oocyte index (FOI), defined as less than 50% in previous cycles despite normal AMH levels, present a unique challenge in assisted reproductive technology (ART) settings. The FOI, which reflects the efficiency of follicular development in producing mature oocytes, serves as a crucial predictor of treatment success in ART cycles. Although these patients have normal AMH levels, commonly used as an indicator of ovarian reserve, they demonstrate suboptimal follicular development and oocyte yield. This suggests potential deficiencies in follicular maturation or oocyte quality. Consequently, managing these patients necessitates tailored strategies aimed at optimizing follicular development and enhancing oocyte quality to improve treatment outcomes (70, 71).

One effective strategy for managing patients with a history of low follicle-to-oocyte index (FOI) is to consider incorporating recombinant human luteinizing hormone (r-hLH) supplementation from the outset of ovarian stimulation protocols or as a rescue option if identified late in the cycle. This approach aligns with the “two-cell, two-gonadotropin” theory, which posits that FSH and LH play critical roles in stimulating the two main cellular components of the ovary: granulosa cells and theca cells, respectively. This stimulation is essential for the secretion of ovarian steroids. FSH is responsible for regulating the proliferation of granulosa cells and promoting estradiol (E2) synthesis, while LH stimulates androgen production by theca cells (31, 72). Although FSH can initiate follicular growth independently, the absence of LH activity results in suboptimal follicular development (73). This inadequacy is attributed not only to the lack of androgen substrates for aromatization but also to the absence of LH direct effects. LH is vital for several ovarian functions: (i) enhancing follicular recruitment by increasing FSH receptor expression in granulosa cells; (ii) promoting follicular maturation through the recruitment of local growth factors; (iii) facilitating the completion of meiosis and the extrusion of the first polar body; and (iv) inducing decidualization of endometrial stromal cells, which is crucial for embryo implantation (74–78). Both the direct effects of LH and androgen production can be impacted by ovarian aging (31). In women undergoing controlled ovarian stimulation (COS) for medically assisted reproduction (MAR), endogenous LH typically supports follicle recruitment. However, women over the age of 35 often experience diminished LH activity, resulting in lower androgen and estrogen levels within the follicular fluid (79). In this context, the supplementation of r-hLH during COS has been suggested to be more effective than high-dose r-hFSH alone in improving clinical pregnancy rates among women of advanced reproductive age (80, 81). However, the effectiveness of co-treatment with r-hFSH and r-hLH in these patients remains a topic of debate, and there is currently no consensus on the appropriate indications for LH supplementation (82).

##### 3.1.2.3 Statement 4

Monitoring levels of follicle-stimulating hormone (FSH), luteinizing hormone (LH), progesterone (P4), and estradiol (E2) at the onset and throughout the cycle facilitates timely adjustments to medication dosages and treatment protocols. This approach is crucial for optimizing patient outcomes in assisted reproductive technology, as it allows for individualized management based on the patient’s hormonal responses. The panel achieved a consensus, with 61.11% agreeing on the importance of this monitoring strategy.

##### 3.1.2.4 Discussion

Monitoring levels of follicle-stimulating hormone (FSH), luteinizing hormone (LH), progesterone (P4), and estradiol (E2) at the start and throughout the menstrual cycle is vital in ART cycles. This monitoring enables timely adjustments in medication dosages and treatment protocols. FSH and LH are essential gonadotropins that play a significant role in regulating follicular development and ovulation, making their assessment critical for evaluating ovarian function and response to stimulation (83–85). Measuring FSH and LH levels at the beginning of the cycle allows clinicians to establish baseline hormonal profiles and detect any underlying hormonal imbalances or dysfunctions that could impact the ovarian response to stimulation. Furthermore, ongoing monitoring of FSH and LH levels throughout the cycle provides real-time insights into follicular growth and maturation, empowering clinicians to make timely adjustments to medication dosages. This proactive approach helps optimize follicular development and prevent premature luteinization (86, 87).

The panel agreed that monitoring progesterone (P4) and estradiol (E2) levels throughout the ART cycle is crucial for evaluating both follicular development and endometrial receptivity. P4 levels increase during the luteal phase of the menstrual cycle, indicating the formation of the corpus luteum and the preparation of the endometrium for embryo implantation. Tracking P4 levels throughout the cycle ensures adequate luteal phase support and may highlight the need for supplementation in cases of luteal phase deficiency. E2, the primary form of estrogen, is vital for regulating follicular development and endometrial proliferation. Monitoring E2 levels offers valuable insights into how well ovarian follicles respond to gonadotropin stimulation and aids in predicting ovulation timing (88–90).

The panel also emphasized that making timely adjustments to medication dosages and treatment protocols based on hormonal monitoring data is essential for optimizing ovarian response and improving treatment outcomes in ART cycles. For instance, if FSH levels are found to be inadequate at the start of the cycle, clinicians may opt to increase the dosage of gonadotropins to promote adequate follicular development. Conversely, if LH levels rise prematurely during the follicular phase, suggesting a risk of premature luteinization, clinicians may need to modify medication dosages or incorporate GnRH agonists or antagonists to prevent ovulation before proper follicular maturation occurs (91–93). Similarly, monitoring P4 and E2 levels throughout the cycle is crucial for evaluating luteal phase support and endometrial receptivity. If P4 levels are found to be low or do not rise adequately during the luteal phase, clinicians may need to supplement with exogenous progesterone to facilitate embryo implantation and support early pregnancy. Furthermore, tracking E2 levels offers valuable insights into follicular development and aids in predicting the timing of ovulation, enabling precise scheduling of procedures like oocyte retrieval (94–96).

##### 3.1.2.5 Statement 5

The panel reached a consensus that to enhance the identification of hypo-responders, it is essential to incorporate the POSEIDON criteria, along with the patient’s BMI, ethnicity, and basal FSH levels, when determining the initial doses of stimulating medications (77.78% agreed).

##### 3.1.2.6 Discussion

To improve the identification of hypo-responders in ART cycles, a comprehensive approach that integrates the POSEIDON criteria with the patient’s BMI, ethnicity, and basal FSH levels is recommended for tailoring treatment strategies. The POSEIDON criteria provide a valuable framework for classifying patients based on their ovarian reserve and likelihood of responding to stimulation. By considering factors such as age, ovarian biomarkers, and previous responses to treatment, clinicians can better identify individuals at risk for suboptimal ovarian stimulation outcomes. Implementing the POSEIDON criteria in clinical practice facilitates a more personalized approach to ovarian stimulation protocols, ensuring that hypo-responders receive targeted interventions aimed at optimizing treatment success (97, 98).

In addition to the POSEIDON criteria, patient-specific factors such as BMI and ethnicity are critical in determining ovarian response to stimulation. Obesity, characterized by a high BMI, is linked to altered hormonal profiles and impaired ovarian function, resulting in diminished responsiveness to FSH stimulation. By considering BMI, clinicians can make necessary adjustments to medication dosages and treatment protocols, optimizing ovarian response in obese patients (34, 99). Similarly, ethnicity has been shown to affect ovarian response to stimulation, with genetic predispositions and variations in hormonal profiles contributing to differences in treatment outcomes among ethnic groups. Incorporating ethnicity into treatment decision-making allows clinicians to customize ovarian stimulation protocols to account for ethnic-specific variations in ovarian response, ultimately enhancing the likelihood of successful outcomes (36).

Basal FSH levels are a critical predictor of ovarian response to stimulation and can help identify patients at risk of hypo-response. Elevated basal FSH levels indicate decreased ovarian reserve and reduced responsiveness to stimulation, highlighting the need for adjustments in medication dosages and treatment protocols to optimize outcomes (100, 101). The current accepted cut-off for normal basal FSH levels is up to 10 IU, but this may not sufficiently differentiate between various types of ovarian responders. Therefore, it is essential to consider redefining the ranges of basal serum FSH to enhance predictions of normal ovarian responses. Additionally, variations in FSH levels across cycles can provide valuable insights, allowing for more personalized treatment approaches and improved management of patients’ expectations (102).

#### 3.1.3 Management technique

##### 3.1.3.1 Statement 6

Efficient scheduling of patients, along with timely appointments, comprehensive monitoring, and coordination with the patient’s menstrual cycle, is crucial for providing optimal care. These factors collectively enhance the management of treatment protocols and improve overall patient outcomes in ART cycles (94.44% agreement).

##### 3.1.3.2 Discussion

Efficient patient scheduling, timely appointments, thorough monitoring, and alignment with the patient’s menstrual cycle are essential for delivering optimal care in ART settings. The strategic timing of appointments and monitoring throughout the ART cycle significantly influences treatment outcomes and patient satisfaction. By ensuring timely monitoring, clinicians can closely observe ovarian stimulation progress, follicular development, and ovulation induction, facilitating necessary adjustments in treatment protocols. Furthermore, coordinating appointments with the patient’s menstrual cycle optimizes the timing for critical procedures, such as oocyte retrieval and embryo transfer, thereby maximizing the chances of successful conception (103–105).

##### 3.1.3.3 Statement 7

Patients experiencing a severe deficiency of LH and FSH should be administered true LH (r-hLH) or LH-like activity (uhCG) to achieve a serum LH level exceeding 1.2 IU/L. This level is essential for providing adequate LH support for FSH-induced follicular development, as agreed upon by the panel (80.56% in favor, 10% neutral).

##### 3.1.3.4 Discussion

LH is not a reliable marker of ovarian function, as many patients may present with LH levels above the normal range yet still experience functional deficiency. Those with severe deficiencies of LH and FSH represent a unique subset of individuals undergoing ART treatments. In these cases, supplementation with exogenous LH is essential to support FSH-induced follicular development. Recombinant human LH (r-hLH) or LH-like activity agents such as urinary hCG (uhCG) can be administered to achieve serum LH levels greater than 1.2 IU/L, which is deemed necessary for optimal follicular development. This supplementation is vital, as LH plays a critical role in the final stages of follicular maturation, including the induction of steroidogenesis and the triggering of ovulation (106, 107).

The target serum LH level of greater than 1.2 IU/L is supported by evidence indicating that this threshold is crucial for FSH-induced follicular development and optimizing oocyte quality. Studies have shown that inadequate LH support during ovarian stimulation can result in suboptimal follicular development, diminished oocyte quality, and lower pregnancy rates. By supplementing with exogenous LH or LH-like activity to achieve the target serum LH level, clinicians can enhance follicular maturation, improve oocyte quality, and increase the likelihood of successful conception (107, 108). The choice between true LH, such as r-hLH, and LH-like activity, such as urinary hCG (uhCG), is influenced by various factors, including availability, cost, and patient preferences. While r-hLH provides pure LH activity without the risk of antigenic reactions, uhCG offers LH-like activity alongside luteal phase support (9, 10). Consequently, clinicians must evaluate the advantages and disadvantages of each option and tailor treatment strategies to meet individual patient needs (109–111).

##### 3.1.3.5 Statement 8

The selection of a stimulation protocol is highly individualized and flexible (based on the patient’s risk factors). (97,22% agreed) The choice of stimulation protocol in assisted reproductive technology is highly individualized and adaptable, taking into account the patient’s specific risk factors and clinical characteristics. (97.22% agreed).

##### 3.1.3.6 Discussion

The selection of a stimulation protocol in ART treatments is a highly individualized and flexible process that takes into account various patient-specific factors and risk factors. ART protocols encompass a range of options, including gonadotropin-releasing hormone (GnRH) agonist protocols, GnRH antagonist protocols, and mild stimulation protocols, each offering distinct advantages and considerations. The decision regarding which protocol to use is based on a comprehensive assessment of the patient’s ovarian reserve, age, reproductive history, and risk factors, allowing for tailored treatment strategies that optimize outcomes while minimizing risks (112–114).

A primary factor in selecting a stimulation protocol is the patient’s ovarian reserve, assessed through markers like AMH levels, antral follicle count (AFC), and basal FSH levels. Patients with diminished ovarian reserve may benefit from a more aggressive stimulation strategy, such as a GnRH agonist protocol, designed to maximize follicular recruitment and enhance oocyte yield. In contrast, patients with normal ovarian reserve may be better suited for milder stimulation protocols that promote a more physiological response while reducing the risk of ovarian hyperstimulation syndrome (OHSS) and other complications (113).

Age is another crucial factor that influences the choice of stimulation protocol, as ovarian responsiveness tends to decline with advancing age. Older patients may require higher doses of gonadotropins or more aggressive stimulation strategies to achieve adequate follicular recruitment and oocyte yield. Furthermore, reproductive history, including previous ART cycles and their outcomes, can guide the selection of a stimulation protocol. Patients with a history of poor ovarian response or failed cycles may benefit from alternative protocols or adjunctive treatments aimed at enhancing ovarian responsiveness and improving treatment outcomes (115).

##### 3.1.3.7 Statement 9

The consensus among experts is that the timing for introducing antagonist medication should be guided by clinical judgment, taking into account the monitoring of lead follicle development and estradiol (E2) levels, with 86.11% in agreement and 10% neutral.

##### 3.1.3.8 Discussion

The timing of introducing antagonist medication in ART treatments is a crucial decision that demands careful consideration and clinical judgment. GnRH antagonists are frequently employed in ART protocols to prevent premature luteinization and enhance follicular development by inhibiting GnRH’s action on the pituitary gland. This timing is usually guided by monitoring lead follicle development and estradiol (E2) levels, enabling clinicians to tailor treatment protocols and optimize ovarian response accordingly (113, 116).

Lead follicle development pertains to the growth and maturation of the dominant follicle(s) in the ovaries during ovarian stimulation. Monitoring this development involves regular transvaginal ultrasound examinations to assess the size and number of follicles throughout the stimulation cycle. Clinicians track lead follicle development to determine the ideal timing for antagonist administration, aiming to prevent premature ovulation while maximizing the number and maturity of follicles available for retrieval. By closely monitoring lead follicle progression, clinicians can pinpoint the optimal window for initiating antagonist treatment and tailor protocols to each patient’s individual response (117–119).

In addition to monitoring lead follicle development, assessing E2 levels offers crucial insights into ovarian response and follicular maturation during ART treatments. Estradiol, the primary form of estrogen produced by the ovaries, is vital for regulating both follicular development and endometrial proliferation. Tracking E2 levels enables clinicians to evaluate the effectiveness of ovarian stimulation and adjust treatment protocols to optimize follicular growth and maturation. Elevated E2 levels may signal excessive follicular recruitment or an increased risk of OHSS, prompting clinicians to consider administering antagonists to halt further follicular development and reduce the likelihood of complications (120–122).

##### 3.1.3.9 Statement 10

The dual trigger approach, which combines HCG (human chorionic gonadotropin) with another agent such as a GnRH agonist, is frequently preferred to improve egg maturation in patients with hypo-responsiveness. (80.56% agreed).

##### 3.1.3.10 Discussion

The dual trigger approach, which combines hCG (human chorionic gonadotropin) with a GnRH agonist, has become a valuable strategy for enhancing oocyte maturation in patients with hypo-response undergoing ART treatments. This innovative method leverages the synergistic effects of hCG and GnRH agonists, which activate different pathways to facilitate final oocyte maturation and improve the quality of retrieved oocytes. By administering both agents, clinicians can address deficiencies in endogenous LH activity and optimize oocyte maturation, particularly in patients with hypo-response or poor ovarian reserve (123–125). Previous studies have validated the efficacy of the dual trigger approach in improving oocyte maturation and overall treatment outcomes in ART. For instance, a randomized controlled trial compared the dual trigger (hCG + GnRH agonist) to hCG alone for triggering final oocyte maturation in IVF patients. The results demonstrated that the dual trigger approach significantly increased the number of mature oocytes retrieved and enhanced embryo quality compared to hCG alone (126, 127).

##### 3.1.3.11 Statement 11

Decisions regarding whether to proceed with fresh or frozen embryo transfer can be personalized by considering various factors, including embryo quality, the uterine environment, and the preferences of both the patient and healthcare provider. (83.33% agreed).

##### 3.1.3.12 Discussion

The decision between fresh and frozen embryo transfer is influenced by several factors, including embryo quality, the uterine environment, and the preferences of both the patient and healthcare provider (128–130). Embryo quality is a critical determinant of success in both fresh and frozen embryo transfer cycles. High-quality embryos, characterized by optimal morphology and developmental potential, have a greater likelihood of successful implantation and pregnancy. Therefore, assessing embryo quality through comprehensive morphological evaluations and advanced techniques such as time-lapse imaging and preimplantation genetic testing can guide the timing of embryo transfer. In instances where embryos demonstrate excellent quality and developmental potential, fresh embryo transfer may be favored to enhance the chances of successful implantation and pregnancy (130, 131). Conversely, when embryo quality is suboptimal or there are concerns regarding the uterine environment, frozen embryo transfer (FET) may provide notable advantages. FET enables the selection of the most viable embryos for transfer following additional culture and selection processes, potentially increasing the likelihood of successful implantation and pregnancy. Additionally, FET allows for the optimization of the uterine environment through controlled ovarian stimulation and endometrial preparation, which may lead to improved outcomes compared to fresh transfer cycles (130, 131).

Patients with reduced ovarian reserve may benefit from preimplantation genetic testing for aneuploidy (PGT-A) to select euploid embryos that possess the correct number of chromosomes for transfer. Transferring euploid embryos identified through PGT-A during a frozen embryo transfer (FET) cycle is recommended to enhance treatment outcomes. FET cycles facilitate the synchronization of embryo transfer with the optimal receptivity of the endometrium and enable the selection of the most viable embryos for transfer. This approach ultimately improves the chances of successful implantation and pregnancy (132, 133).

##### 3.1.3.13 Statement 12

The optimal progesterone level during the luteal phase is generally considered to be higher in fresh embryo transfer cycles, exceeding 50 ng/mL, compared to frozen embryo transfer cycles, where an adequate level is around 10 ng/mL. (55.56% agreed)

##### 3.1.3.14 Discussion

The optimal progesterone level during the luteal phase is a critical factor for successful embryo implantation and the maintenance of pregnancy, particularly in assisted reproductive technology (ART) treatments. Progesterone is essential for preparing the endometrium for embryo implantation and for sustaining a uterine environment that supports pregnancy. However, the ideal progesterone level may differ between fresh and frozen embryo transfer cycles, with specific thresholds recommended for each scenario. In fresh cycles, progesterone levels typically need to exceed 50 ng/mL to enhance implantation success, while in frozen cycles, a level around 10 ng/mL is considered adequate (134, 135).

In fresh embryo transfer cycles, higher progesterone levels (greater than 50 ng/mL) during the luteal phase have been linked to improved implantation and pregnancy outcomes. Elevated progesterone levels enhance endometrial receptivity, facilitating embryo implantation and leading to increased success rates in achieving clinical pregnancies. Thus, maintaining optimal progesterone levels in fresh cycles is crucial for maximizing the chances of successful embryo implantation and subsequent pregnancy (136, 137). In contrast, frozen embryo transfer (FET) cycles can achieve successful implantation and pregnancy with lower progesterone levels, typically around 10 ng/mL, during the luteal phase. Unlike fresh cycles, where ovarian stimulation may result in supraphysiological progesterone levels, FET cycles involve controlled hormonal preparation of the endometrium, which does not necessitate as high progesterone levels for successful implantation. Therefore, the optimal progesterone threshold in FET cycles is lower compared to fresh cycles, reflecting the distinct physiological conditions and treatment strategies involved (138, 139).

##### 3.1.3.15 Statement 13

A personalized approach to luteal phase support in ART treatments may involve a combination of various progesterone administration routes, including vaginal, intramuscular, oral, or rectal options. (88,89% agreed)

##### 3.1.3.16 Discussion

A personalized approach to luteal phase support is crucial in ART treatments to optimize endometrial receptivity and facilitate embryo implantation and early pregnancy. Luteal phase support usually involves administering exogenous progesterone to supplement the body’s natural progesterone production and maintain adequate hormonal levels during this critical period. However, the optimal route of progesterone administration may differ based on individual patient characteristics, preferences, and specific treatment protocols (140, 141).

One key component of personalized luteal phase support is selecting the most appropriate route of progesterone administration for each patient. Several options are available, including vaginal, intramuscular, oral, and rectal routes, each offering distinct advantages and considerations. Vaginal progesterone is frequently preferred due to its ease of use, high bioavailability, and minimal systemic side effects. Research has shown that vaginal progesterone is comparably effective to intramuscular progesterone in supporting the luteal phase and improving pregnancy outcomes in ART treatments (140, 142). Intramuscular progesterone injections, while less convenient than vaginal administration, provide the benefit of consistent and controlled delivery of progesterone, ensuring optimal hormone levels throughout the luteal phase. This route is often favored when vaginal administration is contraindicated or when higher doses of progesterone are necessary. However, intramuscular injections may come with the downside of injection site reactions and discomfort, prompting some patients to prefer alternative methods of administration (143).

Oral and rectal progesterone formulations are less commonly utilized but can be suitable alternatives for patients who prefer non-invasive routes of administration or who have difficulty with vaginal or intramuscular progesterone. Oral progesterone is available in capsule or tablet form and is absorbed through the gastrointestinal tract, while rectal progesterone suppositories are administered into the rectum for absorption via the rectal mucosa. Although oral and rectal progesterone may exhibit lower bioavailability compared to vaginal or intramuscular routes, they remain viable options for luteal phase support in select patients (140, 144).

##### 3.1.3.17 Statement 14

Recombinant human LH exhibits higher dissociation rates from receptor binding sites and has a shorter terminal half-life of approximately 10 hours, compared to hCG, which has a terminal half-life of 28 to 31 hours. Furthermore, the signaling pathways activated by gonadotropins differ: recombinant human LH primarily mediates proliferative and anti-apoptotic signals, while hCG is associated with a higher steroidogenic potential and pro-apoptotic activity *in vitro*. (77.22% agreed)

##### 3.1.3.18 Discussion

The differences between r-hLH and hCG have important implications for their application in ART treatments and luteal phase support. A key distinction lies in their pharmacokinetic properties, including receptor dissociation rates and terminal half-lives. Recombinant human LH demonstrates higher dissociation rates from receptor binding sites compared to hCG, leading to shorter receptor occupancy and a more rapid decline in hormonal activity. Specifically, the terminal half-life of r-hLH is approximately 10 hours, whereas hCG has a longer terminal half-life, ranging from 28 to 31 hours (110, 145, 146).

The pharmacokinetic differences between recombinant human LH (r-hLH) and hCG have important implications for their clinical application in ART treatments, especially regarding luteal phase support. Traditionally, hCG has been favored for luteal phase support due to its longer half-life and sustained hormonal activity. However, r-hLH may present certain advantages in specific clinical scenarios. Its shorter half-life enables more precise control over hormonal levels and allows for a quicker cessation of luteal phase support when necessary. Furthermore, the reduced duration of hormonal activity with r-hLH may lower the risk of prolonged luteal phase support and its associated complications, such as ovarian hyperstimulation syndrome (OHSS) (110, 146). Additionally, the downstream effects of gonadotropin signaling differ between recombinant human LH (r-hLH) and hCG, leading to their distinct physiological actions. Research indicates that r-hLH primarily activates signaling pathways associated with proliferation and anti-apoptotic effects, potentially enhancing endometrial receptivity and embryo implantation. Conversely, hCG is characterized by its high steroidogenic potential and pro-apoptotic activity *in vitro*, which may impact ovarian function and endometrial receptivity (110, 111, 146).

##### 3.1.3.19 Statement 15

For patients with hypo-response who have experienced previous unsuccessful IVF attempts, the use of recombinant human follicle-stimulating hormone (r-hFSH) in combination with recombinant human luteinizing hormone (r-hLH) in the second IVF cycle may be beneficial. (80.56% agreed)

##### 3.1.3.20 Discussion

Patients who experience hypo-response during previous unsuccessful IVF attempts may benefit from a personalized treatment approach that incorporates the use of recombinant human FSH (r-hFSH) and recombinant human LH (r-hLH) in subsequent IVF cycles. Hypo-response, characterized by inadequate ovarian follicular development and a suboptimal response to gonadotropin stimulation, can significantly hinder the success of IVF treatments. In such cases, adding r-hLH to the ovarian stimulation protocol has been suggested as a strategy to enhance follicular development and improve oocyte quality, ultimately increasing the chances of successful embryo implantation and pregnancy (147, 148). Numerous studies have investigated the efficacy of combining r-hFSH and r-hLH in IVF protocols for patients with hypo-response, yielding promising results. These studies have shown improvements in follicular development, oocyte yield, and embryo quality following the addition of r-hLH to ovarian stimulation regimens. Additionally, meta-analyses and systematic reviews have reported higher clinical pregnancy rates and live birth rates in patients receiving combined r-hFSH/r-hLH treatment compared to those treated with r-hFSH alone, particularly among patients with hypo-response (147, 149).

##### 3.1.3.21 Statement 16

Supplementation with recombinant human luteinizing hormone during controlled ovarian stimulation is considered a valuable option for patients exhibiting either predictable or unexpected poor or suboptimal ovarian responsiveness to follicle-stimulating hormone (FSH). This approach is particularly relevant for individuals who meet the Bologna criteria and those classified under the POSEIDON framework. A strong consensus supports the use of r-hLH in these patient populations, with 88.89% of experts in agreement on its potential benefits.

##### 3.1.3.22 Discussion

The incorporation of recombinant human luteinizing hormone (r-hLH) supplementation into controlled ovarian stimulation (COS) protocols presents a promising strategy for patients demonstrating predictable or unexpected poor or suboptimal ovarian responsiveness to follicle-stimulating hormone (FSH). This is particularly relevant for individuals who align with the Bologna criteria or the POSEIDON classification. The POSEIDON classification, which is a relatively recent development, categorizes patients with poor ovarian responsiveness to FSH into two primary groups: “unexpected poor/suboptimal responders” (Groups 1 and 2) and “expected poor/suboptimal responders” (Groups 3 and 4). These classifications are based on a combination of age and ovarian reserve markers (98, 150, 151). A recent systematic review and meta-analysis suggest that adding r-hLH may offer significant benefits for women in POSEIDON Groups 1 and 2, who, despite having well-preserved ovarian reserve markers, demonstrate an unexpected poor or suboptimal response to r-hFSH (82).

Humaidan et al. recently conducted the largest randomized controlled trial (RCT) involving 939 patients classified as poor prognosis, meeting both the ESHRE Bologna criteria and the POSEIDON Group 4 criteria. This study compared a fixed daily dose of 300 IU of recombinant human FSH (r-hFSH) combined with 150 IU of recombinant human LH (r-hLH) to 300 IU of r-hFSH alone. While there were no significant differences in live birth rates (LBR) between the two groups, a *post-hoc* analysis indicated that patients with moderate to severe poor ovarian response (POR) experienced significantly higher LBR and lower total pregnancy loss when supplemented with 150 IU of r-hLH (152). Furthermore, two recent systematic reviews suggest that r-hLH supplementation is beneficial for women with hypo-response, particularly those aged 36 to 39, thus supporting its potential use in patients classified under POSEIDON Group 4 (80, 153).

##### 3.1.3.23 Statement 17

For patients under 35 years of age with an antral follicle count (AFC) greater than 5 and an AMH level above 1.2 ng/ml, using only recombinant human FSH (r-hFSH) may be sufficient during the first IVF cycle. However, incorporating standard doses of recombinant human LH (r-hLH), such as 150 IU per day, is not detrimental and may help prevent unexpected hypo-response, even in younger patients. (72.22% agreed)

##### 3.1.3.24 Discussion

For patients under 35 years of age with an antral follicle count (AFC) greater than 5 and an AMH level above 1.2 ng/ml, initiating controlled ovarian stimulation (COS) with only recombinant human FSH (r-hFSH) is generally regarded as adequate for the first cycle. These indicators typically reflect good ovarian reserve, suggesting that such patients are likely to respond favorably to ovarian stimulation with r-hFSH. Nevertheless, incorporating recombinant human LH (r-hLH) at standard doses (e.g., 150 IU per day) may be a beneficial strategy to mitigate the risk of unexpected hypo-response, even in this relatively low-risk population (147). The study analyzed a real-life cohort of 1,470 women who underwent IVF, categorizing them based on their ovarian response to controlled ovarian stimulation (COS) as poor, suboptimal, or normal. Participants received either recombinant human FSH (r-hFSH) alone or a combination of r-hFSH and recombinant human LH (r-hLH), with the primary outcome measured being the cumulative live birth rate (cLBR). The overall cLBR was higher in the group receiving r-hFSH alone (29.3%) compared to the combination group (22.2%). However, when stratified by ovarian responsiveness, the cLBR was comparable between the two groups among poor and suboptimal responders, indicating that r-hLH supplementation did not adversely affect outcomes, despite these patients having poorer baseline characteristics. Notably, patients receiving r-hLH were generally older and exhibited worse ovarian reserve markers than those treated with r-hFSH alone. This finding suggests that r-hLH supplementation may be advantageous for patients with less favorable prognostic indicators. The study concludes that while r-hFSH alone may suffice for many patients, incorporating r-hLH could help ensure adequate follicular development and potentially enhance outcomes in those with suboptimal or poor response profiles (154).

##### 3.1.3.25 Statement 18

In patients with severe deficiencies in FSH and LH, treatment with recombinant human FSH (r-hFSH) and recombinant LH (r-LH) has been shown to be more cost-effective than human menopausal gonadotropin (hMG). Additionally, this combination therapy is associated with significantly higher pregnancy rates and a shorter time-to-pregnancy. (66,67% agreed)

##### 3.1.3.26 Discussion

In patients with severe deficiencies in gonadotropins, the use of recombinant human FSH (r-hFSH) and recombinant LH (r-hLH) ensures precise dosing and consistent biological activity, which are critical for achieving effective treatment outcomes (30). Research has shown that treatment protocols involving r-hFSH and r-hLH result in higher pregnancy rates compared to human menopausal gonadotropin (hMG). A retrospective analysis involving 999 patients with poor prognoses (defined as an antral follicle count [AFC] of less than 11 and anti-Müllerian hormone [AMH] levels below 1.1 ng/ml) evaluated the long down-regulation protocol and compared a regimen of r-hLH and r-hFSH to hMG. The results indicated that the r-hLH and r-hFSH combination led to a higher clinical pregnancy rate per initiated cycle (12.5% vs. 8.1%, P < 0.02) (155). Notably, this benefit was even more pronounced in patients with an AFC of less than 4, showing pregnancy rates of 10.2% compared to 1.5% with hMG (P < 0.01). This superior performance is attributed to the purity and specific activity of recombinant products, which lack the variability found in urinary-derived hMG preparations (30, 156).

Moreover, the cost-effectiveness of r-hFSH and r-hLH therapy is significant. While the upfront costs of recombinant hormones may be higher than those of human menopausal gonadotropin (hMG), the enhanced pregnancy rates and shorter time-to-pregnancy lead to lower overall treatment costs. This is primarily because fewer treatment cycles are required to achieve pregnancy, which not only alleviates the financial burden on patients but also minimizes the emotional and physical strain associated with undergoing multiple treatment cycles (157).

##### 3.1.3.27 Statement 19

In patients with a low prognosis, the combination of r-hFSH and r-hLH treatment enhance the ongoing pregnancy rate compared to treatment with r-hFSH alone. (69.44% agreed).

##### 3.1.3.28 Discussion

In patients with low prognosis undergoing controlled ovarian stimulation (COS) for IVF, the addition of recombinant human LH (r-hLH) to r-hFSH treatment has been shown to enhance ongoing pregnancy rates compared to the use of r-hFSH alone. This improvement is particularly significant for patients classified under the POSEIDON or Bologna criteria, which identify individuals with poor ovarian response or other factors that suggest a reduced likelihood of success with standard IVF protocols (154). The rationale for incorporating r-hLH into r-hFSH therapy lies in the synergistic roles these hormones play in follicular development and maturation. While FSH primarily stimulates the growth and development of ovarian follicles, LH is crucial for supporting the final stages of follicular maturation, steroidogenesis, and ovulation. In patients with low prognosis, the ovarian follicles may not respond adequately to FSH alone, making it necessary to supplement with LH to achieve optimal follicular development and increase the chances of a successful pregnancy (154, 158).

The addition of recombinant human LH (r-hLH) to recombinant human FSH (r-hFSH) therapy in patients with low prognosis enhances ovarian response, improves the quality of retrieved oocytes, supports endometrial receptivity, and increases ongoing pregnancy rates. This combined approach provides a significant advantage over r-hFSH alone, offering a more effective and tailored treatment strategy for patients facing challenges in their IVF journey. However, it is essential to recognize the current limitations in the quality of available data supporting these findings. While several studies and clinical trials have suggested the potential benefits of combining r-hFSH and r-hLH, the evidence is not uniformly robust or conclusive (154).

#### 3.1.4 Markers of success

##### 3.1.4.1 Statement 20

The measures of success in ART include the quantity, quality, and maturity of eggs, embryo formation by day 3, blastocyst formation and quality, as well as achieving pregnancy, as reflected in the panel’s consensus (94.44% agreed).

##### 3.1.4.2 Discussion

The initial measure of success in ART is determined by the quantity, quality, and maturity of retrieved oocytes. A higher number of retrieved eggs typically correlates with increased chances of obtaining viable embryos, thereby enhancing the likelihood of successful implantation and pregnancy. However, it is essential to consider not only the number of eggs but also their quality and maturity. Mature eggs (MII oocytes) are significantly more likely to undergo successful fertilization and subsequent embryonic development. Factors such as the patient’s age, ovarian reserve, and the stimulation protocol used all influence oocyte quality. Poor egg quality can lead to lower fertilization rates and higher rates of embryonic arrest, as agreed upon by the panel (159–161). By day 3 of embryonic development, successful fertilization is indicated by the formation of multicellular embryos. Key parameters such as the number of cells and the rate of cell division are critical; embryos that reach the 6-8 cell stage by this time are generally considered to be of good quality. Embryo grading systems that evaluate factors like cell number, symmetry, and the degree of fragmentation offer valuable insights into the viability and potential of the embryos to progress to the blastocyst stage and beyond (162). The formation of blastocysts by days 5 to 6 is another critical milestone in embryonic development. This transition from a multicellular embryo to a blastocyst, characterized by the formation of a fluid-filled cavity and differentiation into the inner cell mass and trophectoderm, serves as a strong indicator of embryo viability. High-quality blastocysts are linked to increased implantation and pregnancy rates. Criteria for assessing blastocyst quality include the expansion of the blastocoel, the appearance of the inner cell mass, and the overall quality of the trophectoderm cells (163).

Ultimately, the most definitive measure of success in ART is the achievement of pregnancy. This encompasses biochemical pregnancy, confirmed by elevated hCG levels; clinical pregnancy, verified through ultrasound visualization of a gestational sac; and ongoing pregnancy rates, which refer to pregnancies progressing beyond the first trimester. Among these, the ongoing pregnancy rate is regarded as the gold standard outcome for ART success, as it reflects the culmination of all prior measures into a sustained and healthy pregnancy (164).

### 3.2 Patients with AMA

#### 3.2.1 Profile at presentation

##### 3.2.1.1 Statement 1

Advanced maternal age (≥35 years) can be divided into three distinct age groups: 35-39, 40-43, and over 43. As maternal age increases, the likelihood of obtaining a euploid embryo decreases. This decline in the chances of euploidy is associated with various factors, including the age-related deterioration of oocyte quality and an increase in chromosomal abnormalities. Thus, the age of the patient is a critical consideration in reproductive outcomes (88,89% agreed).

##### 3.2.1.2 Discussion

Advanced maternal age (AMA), generally defined as age 35 and older, significantly impacts reproductive outcomes and can be categorized into three specific age groups: 35-39, 40-43, and over 43 years. The likelihood of successful embryo development diminishes progressively with advancing age, presenting unique challenges and considerations for each subgroup (165, 166). For women aged 35-39, fertility begins to decline, but many can still conceive relatively easily compared to older age groups. This age bracket marks the onset of more noticeable decreases in ovarian reserve and oocyte quality, yet many women remain capable of achieving pregnancy, often with the assistance of ART. Success rates for IVF in this age group are significantly higher than in older women, owing to the relatively better quality and quantity of available eggs. However, even within this group, the risks of chromosomal abnormalities increase, necessitating careful monitoring and, in some cases, genetic screening to ensure healthy embryo development (167, 168).

As women enter the 40-43 age group, fertility declines more sharply. Ovarian reserve significantly decreases, and the proportion of aneuploid eggs increases, markedly impacting both natural conception and ART success rates. The likelihood of achieving pregnancy per IVF cycle drops significantly in this age bracket. Studies indicate that the live birth rate per IVF cycle for women aged 40-42 can be less than half that of women under 35. The heightened prevalence of miscarriages and genetic abnormalities in embryos necessitates a more rigorous approach to embryo selection, often involving preimplantation genetic testing (PGT) to identify euploid embryos. Despite these interventions, cumulative success rates remain lower compared to younger women, highlighting the biological challenges faced in this age range (167, 169).

For women over the age of 43, the challenges become even more pronounced. Ovarian reserve is critically low, significantly reducing the likelihood of retrieving healthy eggs. The live birth rates for women in this age group using their own eggs are exceedingly low, often leading to the consideration of alternative options, such as egg donation. Success rates for IVF cycles using autologous oocytes in women over 43 typically fall below 5% per cycle. This sharp decline is due to both the quantity and quality of the remaining oocytes, many of which are chromosomally abnormal. As a result, many clinics may recommend egg donation as a more viable route to achieving pregnancy, as donor eggs from younger women generally yield higher success rates (169, 170).

##### 3.2.1.3 Statement 2

Both very high and very low BMI significantly influence the distribution of medication throughout the body and the ovarian response to gonadotropins (94.44% agreed).

##### 3.2.1.4 Discussion

It was agreed that BMI plays a crucial role in influencing the pharmacokinetics of medications, including those used in fertility treatments. Both very high and very low BMI can significantly affect the distribution of medications throughout the body, ultimately impacting the ovarian response to gonadotropins, which are essential for assisted reproductive technologies (171). In individuals with high BMI (obesity), increased adipose tissue can sequester lipophilic drugs, leading to reduced bioavailability and efficacy. As a result, obese women often require higher doses of gonadotropins due to altered drug distribution and clearance rates. However, even with these increased dosages, the ovarian response in obese women is frequently suboptimal, characterized by fewer retrieved oocytes and lower embryo quality. Additionally, obesity is linked to elevated estrogen levels, which can negatively affect the hypothalamic-pituitary-ovarian axis and diminish the effectiveness of exogenous gonadotropins (34, 172, 173).

Conversely, low BMI (underweight) can lead to reduced drug reservoirs, resulting in rapid drug clearance and potentially subtherapeutic plasma levels. Very low BMI is often associated with hypothalamic amenorrhea, a condition characterized by disrupted GnRH secretion due to energy deficiency and stress. This disruption results in insufficient production of LH and FSH, both of which are crucial for follicular growth and ovulation. Consequently, underweight women may exhibit diminished ovarian reserve and a poor response to ovarian stimulation, highlighting the need for individualized treatment protocols (34, 171, 172).

#### 3.2.2 Diagnostic tools

##### 3.2.2.1 Statement 3

The primary diagnostic indicators for assessing reproductive health include the patient’s previous history and response to treatment, ovarian reserve, follicular count, genetic testing, receptor polymorphisms, and serum levels of AMH. These factors collectively contribute to a comprehensive evaluation of a patient’s fertility status (94.44% agreed).

##### 3.2.2.2 Discussion

Diagnosing and optimizing fertility treatments necessitates a multifaceted approach that integrates various diagnostic indicators to tailor the most effective interventions for each patient. A patient’s previous history and response to treatment provide critical insights into their fertility challenges and the effectiveness of past interventions. This historical data enables clinicians to identify patterns, such as recurrent implantation failures or poor ovarian response, and to adjust treatment protocols accordingly. For example, a history of inadequate response to standard gonadotropin doses may lead clinicians to consider alternative stimulation protocols or higher gonadotropin doses in subsequent cycles (174).

Assessing ovarian reserve is crucial for evaluating a woman’s reproductive potential. Ovarian reserve refers to both the quantity and quality of a woman’s remaining oocytes, which can be assessed using various methods. A higher antral follicle count (AFC) typically indicates a better response to ovarian stimulation, whereas a lower AFC suggests diminished ovarian reserve and potentially poorer outcomes with standard ART protocols. Anti-Müllerian hormone (AMH) levels correlate with the number of antral follicles and provide a more stable and reliable measure of ovarian reserve compared to other hormonal markers like FSH or estradiol, which can fluctuate during the menstrual cycle (175).

Genetic testing also plays a critical role in diagnosing fertility issues and planning treatments. Genetic anomalies, such as chromosomal translocations or single-gene mutations, can significantly impact fertility and pregnancy outcomes. Preimplantation genetic testing (PGT) allows for the screening of embryos for aneuploidies and specific genetic conditions, enhancing the chances of selecting a viable embryo and reducing the risk of miscarriage or genetic disorders (176). Additionally, considering receptor polymorphisms is an advanced diagnostic tool that aids in customizing treatment. For instance, polymorphisms in the FSH receptor gene have been associated with variations in ovarian sensitivity to FSH, impacting the dosage and protocol required for effective ovarian stimulation. By identifying these genetic variations, clinicians can better predict a patient’s response to gonadotropins and adjust treatment protocols to improve outcomes (177).

##### 3.2.2.3 Statement 4

For patients over 35 years of age with normal ovarian reserve biomarkers, specifically an antral follicle count (AFC) greater than 5 and an AMH level exceeding 1.2 ng/ml, the addition of recombinant human LH (r-hLH) or LH-like activity (u-hCG) supplementation is strongly recommended and may improve pregnancy outcomes. (66.67% agreed).

##### 3.2.2.4 Discussion

As women age, the isoforms of LH shift towards less bioactive forms, particularly becoming more sialylated and less sulfonated (178). This change reduces the effectiveness of circulating gonadotropins, leading to decreased steroidogenesis and compromised ovarian function (31). During the perimenopausal transition, serum gonadotropin levels tend to rise while estradiol (E2) levels decline, resulting in a significant negative correlation between LH receptor (LHCGR) levels and serum LH concentrations (179). Research indicates that decreased LH activity in older women adversely affects androgen production, with circulating androgen levels being significantly lower in women of advanced maternal age (AMA) compared to their younger counterparts (31, 180).

A meta-analysis of studies involving women aged 35 to 40 revealed that co-treatment with recombinant human FSH (r-hFSH) and recombinant human LH (r-hLH) resulted in higher clinical pregnancy rates compared to treatment with r-hFSH alone (181). Younis et al. demonstrated that the addition of r-hLH could effectively compensate for the LH deficiency observed in women over 35 years old (182). Previous randomized controlled trials (RCTs) have also shown that co-treatment with r-hLH and r-hFSH improved live birth and implantation rates in women aged 35 to 39 (183). However, this benefit was not evident in women over 40, likely due to a significant reduction in the proportion of normal euploid embryos in this age group, with no evidence supporting that r-hLH can compensate for this effect (184, 185). The advantages of the r-hFSH:r-hLH treatment for women of advanced maternal age (AMA) may also be associated with LH’s role in oocyte maturation and embryo implantation. LH has been shown to exert an anti-apoptotic effect on cumulus cells and to promote the paracrine signaling necessary for cell expansion and oocyte maturation during folliculogenesis (186).

##### 3.2.2.5 Statement 5

The POSEIDON criteria are widely recognized as a valuable tool for identifying low-prognosis patients in assisted reproductive technology (ART) and planning their treatment strategies. A significant proportion of experts (83.33%) agree that these criteria effectively classify patients based on their ovarian response to stimulation.

##### 3.2.2.6 Discussion

The POSEIDON criteria have emerged as a valuable tool in the field of ART for identifying low-prognosis patients and tailoring treatment strategies accordingly. These criteria classify patients based on their ovarian reserve and response to ovarian stimulation, allowing for more personalized and effective treatment approaches. The POSEIDON criteria take into account factors such as age, AMH levels, AFC, and previous ART outcomes to stratify patients into four groups: Group 1 includes patients with a good prognosis; Group 2 consists of patients with expected poor ovarian response; Group 3 are patients with unexpected poor ovarian response; and Group 4 are composed of patients with a “non-expected” normal ovarian response despite poor ovarian reserve (187). This stratification enables clinicians to design targeted treatment plans that optimize the chances of success for low-prognosis patients, reflecting a consensus among experts regarding its utility in clinical practice. Low-prognosis patients identified using the POSEIDON criteria may benefit from tailored stimulation protocols designed to enhance ovarian response. These approaches could include the use of high-dose gonadotropins, dual stimulation protocols, or the addition of adjuvant treatments such as growth hormone or androgens. Such strategies aim to optimize ovarian stimulation and improve the chances of achieving successful outcomes in patients categorized as low-prognosis (98, 154, 187).

Numerous studies have validated the utility of the POSEIDON criteria in predicting ART outcomes and guiding clinical management. For instance, a retrospective analysis revealed that the POSEIDON criteria effectively predicted ovarian response and clinical pregnancy rates in women undergoing IVF. Furthermore, low-prognosis patients identified by these criteria experienced significantly lower clinical pregnancy and live birth rates compared to those with a good prognosis. This underscores the importance of individualized treatment strategies for this population (188, 189).

#### 3.2.3 Management technique

##### 3.2.3.1 Statement 6

The treatment outcomes of urinary hCG (bioassay-based) and recombinant human LH (mass-based) vary due to differences in their half-lives, bioactivities, and their respective pro- and anti-apoptotic activities. Additionally, the coefficient of variation for *in vivo* bioassays can reach up to 20% for the urinary preparation, highlighting the variability in response (90.56% agreed).

##### 3.2.3.2 Discussion

Urinary hCG, derived from human urine, comprises a mixture of various isoforms of hCG produced by the placenta during pregnancy. These isoforms can differ in bioactivity, potency, and clearance rates, resulting in variable effects on follicular development and ovulation induction in patients undergoing fertility treatments (190, 191). Additionally, the process of purifying hCG from urine can lead to batch-to-batch variability, which may affect its bioactivity and effectiveness in stimulating the final stages of oocyte maturation and ovulation. The coefficient of variation for *in vivo* bioassays of urinary hCG preparations, which measures their biological potency relative to a standard reference, can be as high as 20%. This indicates significant fluctuations in potency and efficacy, underscoring the need for careful consideration of hCG source and preparation in fertility treatments (19, 192).

In contrast, recombinant human LH (r-hLH) is synthesized using DNA technology, ensuring a consistent molecular structure and bioactivity across different production batches. This uniformity in composition and purity facilitates precise dosing and predictable treatment outcomes in ART cycles. Furthermore, the filled-by-mass formulation of recombinant human LH guarantees accurate dosing, with each vial containing a specific amount of biologically active hormone. This minimizes the risk of under- or overdosing during treatment regimens, enhancing the reliability of therapeutic interventions (80, 191).

##### 3.2.3.3 Statement 7

The use of oral contraceptive pills (OCPs) and estrogen priming are considered valid options in COS protocols, particularly for scheduling purposes. OCPs are often employed to regulate the cycle, allowing for greater control over the timing of ovarian stimulation. (80.56% agreed)

##### 3.2.3.4 Discussion

The use of oral contraceptive pills (OCPs) and estrogen priming are valid options in ART protocols, particularly for scheduling purposes. OCPs are commonly employed to synchronize follicular development and facilitate cycle planning. By suppressing endogenous gonadotropin secretion and inducing a withdrawal bleed upon cessation, OCPs enable the coordination of multiple patients’ treatment cycles and the scheduling of procedures such as oocyte retrieval and embryo transfer. Additionally, OCPs help regulate menstrual cycles and reduce the risk of cycle cancellations caused by unexpected deviations in cycle length or timing (193, 194). The use of OCPs, however, invariably leads to an increase in the days of stimulation and an increase in gonadotropin consumption.

Estrogen priming involves administering exogenous estrogen in the early follicular phase of the menstrual cycle to promote endometrial receptivity and optimize ovarian response to gonadotropin stimulation. This approach is particularly beneficial for patients with poor ovarian response or advanced maternal age, where the goal is to enhance follicular recruitment and improve oocyte quality. Estrogen priming may also help mitigate the suppressive effects of elevated baseline follicle-stimulating hormone (FSH) levels on follicular development, thereby enhancing the responsiveness of granulosa cells to exogenous gonadotropins (194, 195).

Several studies have demonstrated the efficacy of OCPs and estrogen priming in improving ART outcomes. For instance, previous investigations found that pretreatment with OCPs was associated with higher clinical pregnancy rates and lower cancellation rates in women undergoing IVF treatment (196, 197). A recent Cochrane review suggested that OCP pre-treatment was associated with a lower rate of live birth among women undergoing ovarian stimulation in antagonist protocols. Other reports indicated that estrogen priming in poor responders led to favorable pregnancy outcomes, highlighting its potential benefits in optimizing ovarian response (194, 198).

##### 3.2.3.5 Statement 8

For patients with AMA, it is generally more effective to use antagonist or short agonist protocols, with a preference for the antagonist protocol (75% agreed).

##### 3.2.3.6 Discussion

For women of AMA, it is generally recommended to employ either GnRH antagonist protocols or short GnRH agonist protocols for ovarian stimulation, with a preference for antagonist protocols (113, 199). GnRH antagonist protocols are favored due to their shorter duration, reduced OHSS risk, and scheduling flexibility. These involve the administration of GnRH antagonists, such as cetrorelix or ganirelix, to immediately suppress premature LH surges, which is especially useful for older patients with diminished ovarian reserve (118, 200).

Short agonist protocols, or “flare” protocols, involve a brief administration of GnRH agonists to trigger an initial release of endogenous gonadotropins (FSH and LH) before transitioning to pituitary suppression. This flare effect can enhance follicular recruitment, making it particularly useful for patients with diminished ovarian reserve, such as those of AMA. It can also be associated with high LH levels in the follicular phase, which may be the subject of concern for their potential detrimental effect on oocyte quality. Furthermore, these protocols carry a higher risk of OHSS, which is a notable concern for older patients with high ovarian reserve, especially those with co-morbidities. Despite the theoretical advantages of flare protocols and antagonist approaches, there is limited high-quality comparative data specifically evaluating their efficacy in AMA patients (113, 200, 201).

##### 3.2.3.7 Statement 9

The recommended dose of recombinant human LH is approximately 150 IU, while the dose of recombinant FSH typically ranges from 300 to 450 IU. However, these values should be adjusted based on individual patient factors, particularly age, to optimize ovarian response and improve clinical outcomes. (69.44% agreed).

##### 3.2.3.8 Discussion

In COS protocols for ART cycles, the recommended dose of r-hLH is generally around 150 IU, with r-hFSH doses ranging from 300 to 450 IU. However, these dosing recommendations are not universally applicable and should be tailored to individual patient characteristics, such as age, basal FSH levels, BMI, antral follicle count (AFC), and previous responses to COS if available. Age is a key determinant, as ovarian reserve and responsiveness to stimulation decline with advanced maternal age (AMA). Consequently, older patients may require higher doses of gonadotropins to promote sufficient follicular development and oocyte maturation (202–204). In such cases, r-hFSH doses between 300 and 450 IU are often necessary to stimulate adequate follicular growth. Depending on the degree of diminished ovarian reserve, as indicated by elevated basal FSH levels, even higher doses may be needed to counteract a poor ovarian response (204, 205).

In addition to AMA, previous responses to COS are crucial in determining the appropriate dosing regimen. Patients with a history of poor response to standard gonadotropin doses may benefit from higher initial doses or the inclusion of additional LH supplementation to enhance follicular development. In such cases, r-hLH doses may be increased to around 225 IU, which has been shown to improve oocyte quality and potentially enhance clinical pregnancy rates (204, 205). Adjusting the dosing based on prior COS outcomes helps optimize stimulation protocols and maximize the chances of successful ART outcomes.

##### 3.2.3.9 Statement 10

The use of recombinant human follicle-stimulating hormone (r-hFSH) in combination with recombinant human luteinizing hormone (r-hLH) is strongly recommended for the first IVF cycle, particularly in patients with hypothalamic hypogonadism and when employing an ultra-long agonist protocol. This approach received broad consensus, with 80.56% of panel members in agreement.

##### 3.2.3.10 Discussion

The rationale for using r-hFSH and r-hLH in women with hypothalamic hypogonadism and in ultra-long agonist protocols is rooted in their ability to offer more precise control over ovarian stimulation, thereby improving treatment outcomes for patients with suboptimal ovarian response or poor ovarian reserve (206–208). These protocols aim to optimize follicular recruitment, increase oocyte yield, and enhance embryo quality in patients with diminished ovarian function or reduced responsiveness to standard stimulation protocols. Supplementing exogenous gonadotropins with r-hFSH and r-hLH allows clinicians to better replicate the physiological environment of follicular development, thereby improving the chances of success in the first IVF cycle (149, 207).

A previous study examined IVF outcomes in a large, real-world population of poor, suboptimal, and normal responders undergoing controlled ovarian stimulation (COS) with either r-hFSH plus r-hLH or r-hFSH alone. Despite worse prognosis indicators in the r-hFSH plus r-hLH group—such as higher age, lower AMH, and lower antral follicle count (AFC)—the study found a reduced oocyte yield, number of mature eggs, and live birth rate (LBR) in this group. However, when r-hLH supplementation was administered, despite significantly older age, patients achieved comparable oocyte yields, produced a similar proportion of mature eggs and top-quality embryos, had a similar number of frozen embryos, and ultimately attained the same clinical LBR per oocyte retrieval as those who received r-hFSH alone (154). This aligns with a systematic review and meta-analysis showing that r-hFSH plus r-hLH co-treatment produces similar clinical outcomes in women of advanced maternal age (82). These findings suggest that LH supplementation may be particularly beneficial for older patients with a poor or suboptimal response to FSH.

##### 3.2.3.11 Statement 11

If progesterone levels on the day of triggering are within the normal range, a fresh cycle may be considered. However, in cases involving preimplantation genetic testing (PGT) or embryo banking, the freeze-all protocol is recommended. This approach is supported by a significant majority, with 88.89% of experts agreeing on its effectiveness in optimizing outcomes in these specific scenarios.

##### 3.2.3.11 Discussion

In assisted reproductive technology, the choice between proceeding with a fresh embryo transfer or adopting a “freeze-all” protocol is influenced by several factors, including follicular progesterone levels, the necessity for preimplantation genetic testing, and specific patient characteristics, such as advanced maternal age. When progesterone levels are within the normal range during the stimulation cycle, a fresh cycle may be deemed viable, permitting immediate embryo transfer after ovarian stimulation and fertilization. However, in scenarios involving PGT-A or embryo banking, the freeze-all protocol is typically recommended. This method entails freezing all viable embryos for transfer in a subsequent cycle, which optimizes the endometrial environment and may enhance implantation rates (209–211).

The preference for a freeze-all strategy in conjunction with preimplantation genetic testing for aneuploidy (PGT-A) is based on several considerations. Elevated progesterone levels during the stimulation cycle have been linked to negative effects on endometrial receptivity, which can adversely affect the success of fresh embryo transfers. By opting to freeze embryos and transferring them in a later cycle with a more controlled endometrial environment, the likelihood of successful implantation and pregnancy may be improved. This protocol is especially beneficial for patients undergoing PGT-A, as it facilitates comprehensive chromosomal analysis and selection of euploid embryos, thereby reducing the risk of miscarriage and enhancing clinical outcomes (212, 213). Despite the prevailing consensus favoring freeze-all protocols, particularly in cases requiring PGT-A, there is a growing discourse on the potential benefits of fresh transfers in specific situations, such as advanced maternal age (AMA) with poor-quality embryos. In these instances, a fresh transfer may be warranted, particularly if embryos are biopsied on day 3 for transfer on day 5. This approach could be advantageous as it circumvents the additional stress and potential epigenetic effects linked to extended culture and biopsy at the blastocyst stage (day 5) (209).

##### 3.2.3.13 Statement 12

Regarding luteal phase support (LPS), the traditional fresh approach is often regarded as the most effective strategy; however, opinions on this matter vary. While 30.56% of experts expressed agreement with this approach, a significant portion of the panel remains uncertain or disagrees.

##### 3.2.3.14 Discussion

Luteal phase support is an essential aspect of IVF protocols, designed to enhance endometrial receptivity and sustain early pregnancy until the placenta takes over hormone production. Traditionally, LPS involves administering progesterone, often in combination with estrogen, after ovulation induction and oocyte retrieval. The approach to LPS can differ significantly between fresh embryo transfer (ET) cycles and frozen embryo transfer (FET) cycles, each presenting its own set of advantages and challenges (214–216). In fresh ET cycles, luteal phase support is typically initiated immediately after egg retrieval and continues through the early stages of pregnancy. This method relies heavily on the body’s response to ovarian stimulation and the subsequent endogenous hormone production to sustain the luteal phase (135, 216).

However, the effectiveness of the “regular fresh approach” remains a topic of debate within the field. While 30% of experts consider it the most effective, an equal percentage disagree, and 40% remain neutral, indicating a lack of consensus. One notable drawback of fresh cycles is the risk of ovarian hyperstimulation syndrome (OHSS), which can be exacerbated by elevated endogenous hormones resulting from ovarian stimulation. Fresh cycles also have a higher risk of ectopic pregnancy compared to frozen embryo transfer (FET) cycles (135, 216–218). In contrast, pregnancy rates tend to be higher with FET cycles, attributed to several factors. Firstly, FET cycles allow for a more controlled and optimized endometrial environment, free from the hormonal fluctuations associated with ovarian stimulation. This control is particularly important for patients who may experience elevated progesterone levels during fresh cycles, which can adversely affect endometrial receptivity (129). Furthermore, FET cycles provide the opportunity for preimplantation genetic testing for aneuploidy (PGT-A), enabling the selection of chromosomally normal embryos and thereby increasing the likelihood of successful pregnancies (217, 219). Additionally, FET cycles significantly reduce the risks associated with fresh transfers, as the ovaries have time to recover before starting a new cycle, thereby lowering the risk of OHSS (217).

##### 3.2.3.15 Statement 13

Vaginal progesterone therapy is widely recognized as the gold standard for luteal phase support following IVF/ICSI procedures. There is consensus that the route of progesterone administration does not significantly affect treatment outcomes, underscoring the flexibility in choosing the most suitable method for individual patients (41.56% agreed).

##### 3.2.3.16 Discussion

Vaginal progesterone therapy has long been considered the gold standard for luteal phase support following IVF and ICSI, primarily due to its direct delivery to the endometrium and favorable side effect profile. While many recognize its efficacy, there is ongoing debate regarding the impact of the route of administration on treatment outcomes. Approximately 40% of experts agree that the route does not influence results, while an equal percentage disagrees (220). The use of vaginal progesterone is prevalent because it achieves high local concentrations of the hormone at the site of action—the endometrium—while minimizing systemic side effects. This administration route bypasses first-pass metabolism in the liver, leading to more stable progesterone levels in the endometrium. It is particularly advantageous in frozen embryo transfer (FET) protocols, where vaginal progesterone can be integrated into natural cycles, modified natural cycles, ovulation induction cycles, or hormone replacement therapy (HRT) cycles. Each of these protocols necessitates careful consideration of luteal phase support to enhance endometrial receptivity and implantation success (220).

Despite its widespread use, vaginal progesterone alone may not suffice for all patients, particularly in frozen embryo transfer (FET) cycles. The intramuscular (IM) route can ensure adequate serum progesterone levels, which are essential for maintaining a supportive luteal phase environment, especially in individuals with suboptimal absorption of vaginal progesterone. Research indicates that IM progesterone typically leads to higher serum progesterone levels compared to vaginal administration alone, potentially contributing to improved implantation and pregnancy outcomes. While vaginal progesterone is favored as the gold standard due to its ease of use and high patient compliance, IM progesterone is often recommended for its superior efficacy in specific patient populations (221, 222). Serum progesterone measurements have been advocated to optimize luteal phase support FET cycles. This highlights the importance of individualized treatment plans that consider each patient’s unique circumstances to optimize reproductive outcomes.

##### 3.2.3.17 Statement 14

There is consensus that the quality of the luteinizing hormone (LH) source significantly affects ovarian response, with this influence being further modulated by the patient’s age (80.56% agreed).

##### 3.2.3.18 Discussion

As women age, the functionality and reserve of their ovaries decline, leading to a reduction in both the quantity and quality of oocytes. This age-related decrease in ovarian reserve is often accompanied by alterations in the hypothalamic-pituitary-ovarian axis, resulting in varying levels of endogenous luteinizing hormone (LH) and follicle-stimulating hormone (FSH) (223). Older women typically demonstrate diminished ovarian sensitivity to gonadotropins, necessitating higher doses and potentially more potent formulations of exogenous LH to elicit an adequate ovarian response. Consequently, the source and quality of LH utilized in controlled ovarian stimulation (COS) protocols can significantly impact the effectiveness of stimulation and the overall outcomes of assisted reproductive technology (ART) procedures (224, 225).

Recombinant human LH and human-derived urinary LH (u-LH) are the two primary sources of exogenous LH available for clinical use. Recombinant human LH offers a high level of purity and consistency, translating to more predictable and effective ovarian stimulation. In contrast, urinary-derived LH, while effective, can exhibit variability in bioactivity due to the complex extraction and purification processes involved. This variability may result in differences in ovarian response among patients, particularly those of advanced maternal age (AMA), who often require more precise dosing to achieve optimal follicular development (110, 111, 148, 181). The purity and consistency of recombinant human LH are particularly advantageous for older patients, who typically exhibit a more attenuated response to ovarian stimulation due to reduced ovarian reserve and altered hormonal profiles. Studies indicate that the use of recombinant human LH in conjunction with recombinant human FSH (r-hFSH) can enhance follicular response and oocyte quality in older women compared to protocols utilizing urinary-derived gonadotropins (82, 111, 181, 185).

#### 3.2.4 Markers of success

##### 3.2.4.1 Statement 15

Markers of success in ART include the live birth rate, pregnancy rate, number of eggs retrieved, number of embryos formed, and the development of blastocysts following treatment administration (94.44% agreed).

##### 3.2.4.2 Discussion

The live birth rate is widely regarded as the most definitive marker of success in Assisted Reproductive Technology (ART), as it directly measures the ultimate objective of fertility treatments: the birth of a healthy baby. This metric encompasses all stages of the ART process, from ovarian stimulation and egg retrieval to embryo transfer and pregnancy maintenance. Research consistently indicates that live birth rates are influenced by several factors, including the patient’s age, ovarian reserve, and the quality of the embryos transferred (226). The pregnancy rate, often referred to as the clinical pregnancy rate, is another crucial indicator of ART success. This metric typically reflects the presence of a gestational sac, with or without a fetal heartbeat, as confirmed by ultrasound, and serves as an early gauge of treatment effectiveness. Although it is not as definitive as the live birth rate, the pregnancy rate offers valuable insights into the initial success of embryo implantation and early embryonic development. Factors influencing pregnancy rates include the quality of the endometrial environment, the synchronization between endometrial and embryo development, and the hormonal support provided during the luteal phase (227).

The number of eggs retrieved during ovarian stimulation serves as a critical intermediate marker of success in Assisted Reproductive Technology (ART), reflecting both the response to gonadotropin administration and the patient’s ovarian reserve. A greater number of eggs retrieved enhances the likelihood of obtaining viable embryos for transfer or cryopreservation, thereby increasing the chances of achieving pregnancy and live birth (8, 228). Following egg retrieval, the number of embryos formed becomes a key indicator of ART success, reflecting the fertilization efficiency and developmental potential of the retrieved oocytes. The presence of multiple high-quality embryos provides options for fresh transfer, future frozen embryo transfer (FET), and preimplantation genetic testing (PGT) to select the most viable embryos (229). The development of blastocysts, which occurs when embryos reach the fifth or sixth day of development, is another significant marker of ART success. Blastocyst development indicates that the embryos have successfully navigated critical early stages, such as cleavage and compaction, demonstrating strong developmental potential. Transferring blastocysts, rather than earlier-stage embryos, is associated with higher implantation rates and improved pregnancy outcomes due to better synchronization with the endometrial environment and the ability to select embryos with enhanced developmental competence (230).

### 3.3 Strengths and limitations

The consensus reflects the collective expertise and experience of experts in the field of ART, offering a comprehensive perspective on managing patients with hypo-response to gonadotropin stimulation and advanced maternal age. By incorporating evidence-based practices, the consensus ensures that its recommendations are grounded in scientific research and clinical experience. It addresses various facets of patient profiling, diagnostic tools, management techniques, and markers of success, providing a holistic framework for optimizing fertility treatment strategies and enhancing patient care. The unanimous agreement on certain recommendations, such as the use of recombinant human LH supplementation and specific markers of success, validates established practices and underscores areas of broad consensus within the ART community. However, the consensus has limitations due to the relatively small number of participants, which may restrict the diversity of perspectives and experiences represented, potentially affecting the generalizability of the recommendations. Individual biases or preferences among participants could also influence the consensus process, leading to discrepancies in opinion and variability in the strength of agreement on certain recommendations. Furthermore, the field of ART is dynamic, with continuous advancements and changes in practice guidelines; thus, the consensus may not fully capture the latest developments or emerging trends, highlighting the need for periodic updates to maintain relevance and accuracy.

## 4 Conclusion

The expert panel reached unanimous agreement on key risk factors predisposing individuals to hypo-response, underscoring the necessity for personalized care that considers patient-specific characteristics such as age, BMI, and medical history. Diagnostic tools, including hormone level monitoring and the incorporation of criteria like POSEIDON, were supported by the majority, highlighting the importance of a comprehensive assessment in treatment planning. There was widespread consensus on the need for individualized stimulation protocols and efficient patient scheduling, emphasizing personalized approaches to enhance treatment outcomes.

Furthermore, the panel unanimously endorsed the use of recombinant human LH supplementation and a tailored approach to luteal phase support. However, opinions varied regarding optimal progesterone levels during the luteal phase and the dual trigger approach, indicating a need for further research and clinical evaluation in these areas. The consensus also emphasized the significance of success markers, such as embryo quality, pregnancy rates, and live birth rates, in assessing treatment efficacy.

Overall, the consensus reflects the complexity of fertility treatment decision-making and reinforces the importance of individualized strategies based on patient-specific factors. By integrating multidisciplinary expertise and evidence-based practices, clinicians can optimize treatment outcomes and improve the success of ART for patients experiencing hypo-response to gonadotropin stimulation and those of advanced maternal age.

## Data Availability

The original contributions presented in the study are included in the article/supplementary material. Further inquiries can be directed to the corresponding author.
